# A comprehensive study on lithium-based reactive hydride composite (Li-RHC) as a reversible solid-state hydrogen storage system toward potential mobile applications†

**DOI:** 10.1039/d1ra03246a

**Published:** 2021-06-30

**Authors:** Fahim Karimi, Philipp Klaus Pranzas, Julián Atillio Puszkiel, María Victoria Castro Riglos, Chiara Milanese, Ulla Vainio, Claudio Pistidda, Gökhan Gizer, Thomas Klassen, Andreas Schreyer, Martin Dornheim

**Affiliations:** Department of Nanotechnology, Institute of Materials Research, Helmholtz-Zentrum HEREON Max-Planck-Straße 1 21502 Geesthacht Germany fahim.karimi2017hh@gmail.com; Department of Physicochemistry of Materials, Consejo Nacional de Investigaciones Científicas y Técnicas (CONICET) y Centro Atómico Bariloche Av. Bustillo km 9500 S.C. de Bariloche Argentina; Department of Metalphysics, Consejo Nacional de Investigaciones Científicas y Técnicas (CONICET) y Centro Atómico Barilo-che Av. Bustillo km 9500 S.C. de Bariloche Argentina; C.S.G.I. & Department of Chemistry, Physical Chemistry Section, University of Pavia Viale Taramelli 16 27100 Pavia Italy; Hitachi High-Tech Analytical Science Finland Finland; European Spallation Source ERIC Box 176 S-22100 Lund Sweden

## Abstract

Reversible solid-state hydrogen storage is one of the key technologies toward pollutant-free and sustainable energy conversion. The composite system LiBH_4_–MgH_2_ can reversibly store hydrogen with a gravimetric capacity of 13 wt%. However, its dehydrogenation/hydrogenation kinetics is extremely sluggish (∼40 h) which hinders its usage for commercial applications. In this work, the kinetics of this composite system is significantly enhanced (∼96%) by adding a small amount of NbF_5_. The catalytic effect of NbF_5_ on the dehydrogenation/hydrogenation process of LiBH_4_–MgH_2_ is systematically investigated using a broad range of experimental techniques such as *in situ* synchrotron radiation X-ray powder diffraction (*in situ* SR-XPD), X-ray absorption spectroscopy (XAS), anomalous small angle X-ray scattering (ASAXS), and ultra/small-angle neutron scattering (USANS/SANS). The obtained results are utilized to develop a model that explains the catalytic function of NbF_5_ in hydrogen release and uptake in the LiBH_4_–MgH_2_ composite system.

## Abstract 2

Superb dehydrogenation/hydrogenation kinetic enhancement of the LiBH_4_–MgH_2_ reactive hydride composite system by addition of NbB_2_ nano-particles as nucleation agents for MgB_2_.

## Graphical abstract

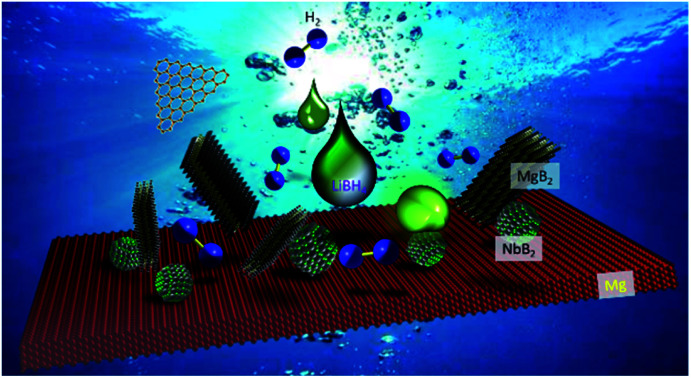

## Introduction

The global energy demand is currently covered mainly by fossil fuels and nuclear power. By following this path, it will be a question of when these limited resources will be exhausted. Moreover, massive exploitation/consumption of these resources can lead to irreversible ecological disasters. Considering these facts, strategies and concepts for clean and sustainable energy solutions are urgently needed. A widely recognized candidate for sustainable and pollutant-free energy conversion is considered to be hydrogen. This is due to its high energy density per mass unit (33.3 kW h kg^−1^) in comparison to natural gas (13.9 kW h kg^−1^) and liquid fossil fuels (12.4 kW h kg^−1^),^1,2^ and because its combustion product is just water: 2H_2_ + O_2_ → 2H_2_O, using a fuel cell. By using sustainable energy sources (such as sun, wind, biomass energy, *etc.*) to produce hydrogen, a renewable energy circuit can be created. The main obstacles hindering hydrogen being used as an energy carrier in a wide variety of industrial applications is its low volumetric energy density (∼0.003 kW h dm^−3^) and its low ignition energy (∼0.02 MJ at hydrogen/air volume ratio ≈ 29%; a spark could ignite it) in comparison to gasoline (∼9 kW h dm^−3^, 0.24 ml gasoline vapor) at standard conditions.^3^ In order to enhance the volumetric energy density of gaseous hydrogen, two alternative approaches are probed so far: (1) gaseous hydrogen is pressurized up to ∼700 bar at 15 °C (1.2 kW h dm^−3^), and (2) gaseous hydrogen is liquefied at cryogenic temperatures (2.4 kW h dm^−3^).^4^ Although these methods do improve the volumetric energy density of gaseous hydrogen, the safety issue remains still challenging. A more convenient alternative approach is to store hydrogen chemically in metal hydrides. This concept not only improves the volumetric energy density of the gaseous hydrogen significantly, but it also avoids the above-mentioned safety issues. Since hydrogen atoms are chemically bonded (stored) in the host metal, an energy barrier has to be overcome before hydrogen can be released. Especially, light metal hydrides are of great interest for hydrogen storage in mobile applications due to their high gravimetric- and volumetric energy densities. In particular, borohydrides of alkaline and alkaline earth metals show exceptionally high gravimetric hydrogen storage capacity. LiBH_4_ has one of the highest volumetric energy densities (4.0 kW h dm^−3^)^5^ and one of the highest gravimetric hydrogen capacities (∼13 wt%).^6^ However, its decomposition starts at temperatures above 700 K according to reaction (1),^5,7,8^ due to its thermodynamic stability (67–74 kJ mol^−1^ H_2_),^9–12^ that is very high relative to the reference stability window (30–50 kJ mol^−1^ H_2_) defined by the DOE.^13^

1 LiBH_4_ → LiH + B + 

<svg xmlns="http://www.w3.org/2000/svg" version="1.0" width="18.545455pt" height="16.000000pt" viewBox="0 0 18.545455 16.000000" preserveAspectRatio="xMidYMid meet"><metadata>
Created by potrace 1.16, written by Peter Selinger 2001-2019
</metadata><g transform="translate(1.000000,15.000000) scale(0.015909,-0.015909)" fill="currentColor" stroke="none"><path d="M80 840 l0 -40 -40 0 -40 0 0 -40 0 -40 40 0 40 0 0 40 0 40 120 0 120 0 0 -40 0 -40 -80 0 -80 0 0 -40 0 -40 80 0 80 0 0 -80 0 -80 -120 0 -120 0 0 40 0 40 -40 0 -40 0 0 -40 0 -40 40 0 40 0 0 -40 0 -40 120 0 120 0 0 40 0 40 40 0 40 0 0 80 0 80 -40 0 -40 0 0 40 0 40 40 0 40 0 0 40 0 40 -40 0 -40 0 0 40 0 40 -120 0 -120 0 0 -40z M720 840 l0 -40 -40 0 -40 0 0 -80 0 -80 -40 0 -40 0 0 -80 0 -80 -40 0 -40 0 0 -80 0 -80 -40 0 -40 0 0 -80 0 -80 -40 0 -40 0 0 -40 0 -40 -40 0 -40 0 0 -40 0 -40 80 0 80 0 0 40 0 40 40 0 40 0 0 80 0 80 40 0 40 0 0 80 0 80 40 0 40 0 0 -40 0 -40 80 0 80 0 0 40 0 40 40 0 40 0 0 -80 0 -80 -40 0 -40 0 0 -40 0 -40 -40 0 -40 0 0 -40 0 -40 -40 0 -40 0 0 -40 0 -40 200 0 200 0 0 40 0 40 -80 0 -80 0 0 40 0 40 40 0 40 0 0 80 0 80 40 0 40 0 0 40 0 40 -40 0 -40 0 0 40 0 40 -120 0 -120 0 0 -40 0 -40 -40 0 -40 0 0 80 0 80 40 0 40 0 0 80 0 80 40 0 40 0 0 80 0 80 -40 0 -40 0 0 -40z"/></g></svg>

H_2_

A partial rehydrogenation of reaction (1) can be achieved only at temperatures above 900 K and at hydrogen pressures of ∼15 MPa,^14,15^ which makes this complex hydride unsuitable for a possible mobile and/or stationary application. An alternative approach that avoids a dramatic degradation in hydrogen capacity and simultaneously reduces the reaction enthalpy is the concept of so-called “reactive hydride composite” (RHC).^16–18^ In this approach, one or more metal hydride is added to the complex metal hydride forming a high hydrogen storage capacity composite system with a lower thermodynamic stability and enhanced reversibility.^12,19^ The reaction-enthalpies of various systems were altered by using the this concept.^20–29^ In this work, MgH_2_ is used to stabilize the dehydrogenation products of LiBH_4_. Indeed, the overall reaction enthalpy of the LiBH_4_–MgH_2_ reactive hydride composite system (Li-RHC) is reduced to 46 kJ mol^−1^ H_2_,^12^ in comparison to the respective values of each single metal hydride (LiBH_4_ (67 kJ mol^−1^ H_2_), MgH_2_ (75 kJ mol^−1^ H_2_)),^5,30^ according to the following reaction path:

2 2LiBH_4_ + MgH_2_ ↔ 2LiH + MgB_2_ + 3H_2_

Experimentally, however, this composite system can take different reaction paths depending on the applied hydrogen backpressures and temperatures.^31^ At applied hydrogen backpressures lower than 3 bar and at temperatures about 400 °C, the system's components decompose independently:

3 2LiBH_4_ + MgH_2_ → 2LiH + Mg + B + 4H_2_

In this case, the overall reaction enthalpy of this composite system is not altered, and it is only partially reversible (due to its possible stable decomposition products such as amorphous boron). Though reaction (2) is thermodynamically more stable compared to reaction (3), independent decomposition of the hydrides is kinetically favored in latter case. By applying hydrogen backpressures greater than 3 bar at temperatures around 400 °C, the decomposition of LiBH_4_ is thermodynamically prevented, however not that of MgH_2_. Thus, after decomposition of MgH_2_ to metallic magnesium, subsequently Mg reacts with LiBH_4_ to form MgB_2_.^31,32^ In this case, the dehydrogenation reaction can be described as follows:^33^

4.1 2LiBH_4_ + MgH_2_ → 2LiBH_4_ + Mg + H_2_

4.2 2LiH + MgB_2_ + 3H_2_

Upon the dehydrogenation reaction, an amount of roughly 11.5 wt% hydrogen is released. The first reaction step proceeds endothermic, while the second reaction step takes place exothermic, which causes a reduction in the overall reaction enthalpy of the system. Hence, the formation of MgB_2_ is crucial to ensure the reversibility of Li-RHC. This is, due to a graphite-like layered structure of MgB_2_, compared to the *closo*-structure of boron (Fig. S1, ESI†).^19^ The rehydrogenation of 2LiH + MgB_2_ proceeds in one step under moderate thermodynamic conditions (50 bar H_2_, 350 °C), according to reaction (5):

5 2LiH + MgB_2_ + 3H_2_ → 2LiBH_4_ + MgH_2_

Although thermodynamics and reversibility of LiBH_4_ can be tuned applying the RHC concept, the de/rehydrogenation reaction kinetics of the system remains rather slow. In this work, the dehydrogenation and rehydrogenation reaction kinetics of this composite system could be significantly improved (∼ 96%) by an addition of a small amount of NbF_5_. The catalytic role of NbF_5_ in enhancing the dehydrogenation/rehydrogenation reaction kinetics of Li-RHC is studied in the following in detail using advanced experimental methods such as small-angle X-ray scattering (SAXS), anomalous small-angle X-ray scattering (ASAXS), small-angle neutron scattering/ultra-small-angle neutron scattering (SANS/USANS), *in situ* synchrotron radiation X-ray powder diffraction (*in situ* SR-XPD), and high-resolution transmission electron microscopy (HR-TEM).

## Experimental

The raw material were purchased from Sigma-Aldrich, (with degree of purity of: LiBH_4_ ≥ 97.0%, MgH_2_ ≥ 90.0%, and NbF_5_ ≥ 98.0%), and the isotope containing materials (Li^11^BH_4_, and ^7^Li^11^BH_4_ with purities of ≥95%) were purchased from KAT-Chem Ltd. All samples were prepared by ball-milled in a Spex 8000 mill/shaker. The Spex 8000 mill/shaker was placed in a glove box (MB-BRAUN, Germany) under a continuously purified argon atmosphere with less than 10 ppm oxygen and moisture, respectively. The milling vial and the milling balls were made of stainless steel. The utilized ball-to-powder ratio was 10 : 1. MgH_2_ was premilled for 5 h before mixing it with LiBH_4_/NbF_5_. Volumetric measurements were performed using the Sievert's type apparatus Hydro Quebec, Canada,^34^ in order to study the dehydrogenation/hydrogenation properties of the composite systems and prepare cycled samples for further measurements. Coupled manometric-calorimetric desorption measurements were performed by connecting a PCT-Pro instrument (Setaram and Hy-Energy, France) with a Sensys high-pressure DSC (Setaram, HP-DSC, France). For each measurement, the mass of the samples was chosen to be about 30 mg. The measurements were performed by heating the samples from room temperature up to 500 °C at 3 °C min^−1^ under a H_2_ pressure of 4 bar. *In situ* SR-XPD measurements were carried out at the powder diffraction beamline D3 at the DORIS III synchrotron storage ring (at DESY in Hamburg, Germany). For *in situ*SR-XPD measurements, samples were loaded into single-crystal sapphire tubes in a glove box filled with argon atmosphere. The sapphire tubes were subsequently mounted in a gas pressure cell.^25^ Thereafter, the cell unit was transferred into the experimental hutch, where it was mounted to a goniometer and subsequently exposed to the synchrotron beam. The incident photon wavelength was set to 0.5 Å. A MarCCD-165 area detector was applied to collect the diffracted intensities. The diffraction intensities from the sample were collected after each 30 seconds, and the sample-to-detector distance (SDD) was kept at about 125 mm. The sample was heated up by a ceramic oven placed underneath the capillary while the sample temperature was measured by a thermocouple positioned close to the sample in the capillary and controlled *via* a PID regulator. The temperature and pressure parameters were set to the same values as they were chosen for the volumetric measurements. The acquired 2D-patterns were further processed to a 1D diffractogram using the Fit2D program,^35^ and the program FindIt (ICSD-database)^36^ was used for phase identification. X-ray absorption spectroscopy (XAS) measurements were performed at the C-beamline at DORIS III. The ideal amount of the samples for the measurements was calculated by using the program XAFSMASS.^37^ Samples were mixed with dry cellulose (∼50 mg) in a mortar and pressed (5 bar) into pellets of 10 mm in diameter. The pellets were placed then in an aluminum sample holder and sealed with Kapton tape (55 µm, in thickness) to avoid sample's oxidation. Niobium was measured in its various oxidation states (metallic Nb, NbF_5_, and NbB_2_) as reference material. All measurements were recorded in transmission mode as well as in fluorescence mode at the K-edge of metallic Nb (18.99 keV). For each sample, three XAS-spectra were acquired. After removing the spikes, the spectra were aligned, calibrated, and subsequently averaged. XAS data processing and analysis were conducted by using the “IFEFFIT” software package.^38^ Anomalous small-angle X-ray scattering (ASAXS) measurements were performed at the beamline B1 at DORIS III (DESY in Hamburg, Germany). All measurements were carried out near the K absorption edge of niobium (18.99 keV) to characterize the Nb-containing structures in the samples upon dehydrogenation/rehydrogenation cycles. Samples were mounted in an aluminum sample holder with a circular hole (5 mm in diameter and a thickness of 0.25 mm). All samples were sealed with Kapton tape to avoid any possible oxidation. The ASAXS intensities were acquired at four different energies at two SDD (*D*_min_ = 885 mm and *D*_max_ = 3585 mm), respectively, to cover the maximum experimental *q*-range available. Here, *q* is the magnitude of the scattering vector defined as: *q* = (4π/*λ*)sin *θ*, where *λ* is the incident X-ray wavelength and 2*θ* is the respective scattering angle. The beamline was equipped with a Pilatus 300 K detector and a Si (311) double crystal monochromator with a wavelength resolution of Δ*λ*/*λ* < 10^−4^. All selected energies with their corresponding anomalous dispersion factors are listed in Table 1 (the calculated dispersion values are based on the theory of Cromer & Lieberman).^39^ Small-Angle Neutron Scattering (SANS) measurements were performed at the SANS-1 instrument at FRM II of Heinz Maier-Leibnitz Zentrum (MLZ, Bavaria, Germany)*.*^40^ The thermal neutrons were from a source with a maximum flux of 8 × 10^14^ n cm^−2^ s^−1^. The incident neutron wavelength could be monochromated in the range of 3.4–30 Å by applying an array of two mechanical velocity selectors with a wavelength resolution of 10% and 6%, respectively. The SDD could be set in the range of 1.1–21 m, with a maximum *q* range of 0.001–2 Å^−1^. For the performed experiments in this work, three sample to detector distances (1.6, 8, 20 m) and two wavelengths (6 Å for 1.6 and 8 m, 12 Å for 20 m) were applied, respectively, to cover the maximum *q*-range available at the high *q* values and to cover a reasonable *q*-range in the low *q* region in order to have a good overlapping with the USANS measurements.

**Table 1:** Selected energies for the ASAXS-measurements at the K-edge of Nb and the corresponding anomalous dispersion factors^41^

#	Energy [eV]	*f*′	*f*″
E1	18 522	−3.097	0.557
E2	18 822	−4.078	0.542
E3	18 924	−5.035	0.542
E4	18 926	−5.068	0.543
E5	18 976	−6.907	0.668

The scattering intensities were measured by using a 128 ^3^He proportional counter detector with an area of 1000 × 1020 mm^2^ with a count rate of 1 MHz. Silverbehenate (AgBe) was measured at a short distance (1.6 m) to calibrate the *q*-axis. A water standard was measured in order to set the scattering intensities onto an absolute scale. All SANS data were processed by using BerSANS software.^42^ In addition to SANS measurements, USANS measurements were carried out to evaluate the larger structures present in the samples. USANS measurements were performed at the BT5-Instrument at the National Institute of Standard and Technology (NIST) in Gaithersburg (Maryland, USA).^43^ TEM characterization was performed on a FEI TECNAI G^2^ machine using 200 kV, point resolution: 0.12 nm, and a field emission gun (FEG). Dark Field imaging technique (DF) was used to distinguish different phases by their diffraction. Size measurements of the particles were performed by means of an interpolated polygon tool from iTEM software,^44^ and the values that took into account were those from mean diameter measurements. High-resolution transmission electron microscopy (HR-TEM) images were obtained with a magnification higher than *M* > 300k×. In HR-TEM images, Fast Fourier Transform (FFT) was performed by Digital Micrograph software^45^ to obtain the diffraction patterns. These were compared with the simulated ones obtained by JEMS software.^46^ Samples for TEM were prepared by dispersing a small amount of powder in hexane and then ultrasonicated the suspension for 10 min. A drop of this suspension was deposited over a commercial copper grid for TEM coated with a Formvar support film. In this procedure, the samples were exposed to air for a short period.

## Results

**Fig. 1:**
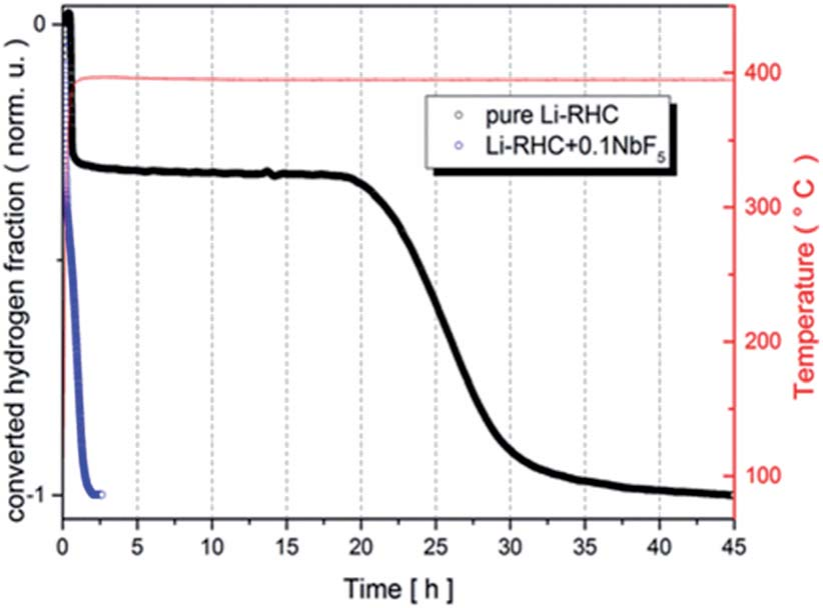
Dehydrogenation reaction kinetics of pure and doped Li-RHC under 4 bar of hydrogen back pressure. The samples were heated up (5 K min^−1^) to about 390 °C and were kept isothermal for the rest of the measurement.

The first dehydrogenation reactions of pure Li-RHC and the doped Li-RHC + 0.1NbF_5_ sample are presented in Fig. 1, where the normalized converted fraction of desorbed hydrogen is plotted over the corresponding elapsed time in hours. The overall dehydrogenation process of the pure Li-RHC system is completed after approximately 45 h. This reaction proceeds in two distinct steps, which are separated by a plateau region. The first reaction step is finished after 0.75 h, followed by the plateau phase, which extends nearly up to 18 h, before the second reaction step starts. The second reaction step is completed after roughly 20 h.

**Fig. 2:**
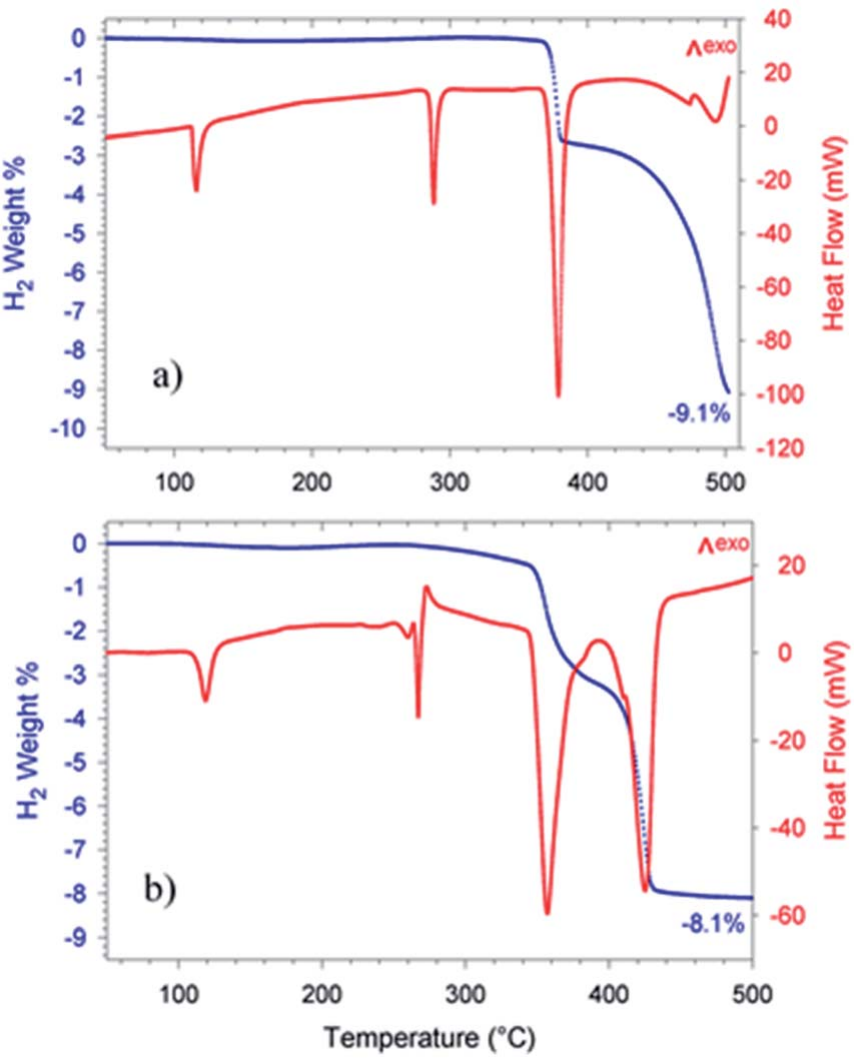
Coupled manometric- DSC measurements of the first dehydrogenation reaction of pure (a) and doped Li-RHC (b) under 4 bar of hydrogen backpressure.

The dehydrogenation reaction of the doped Li-RHC + 0.1NbF_5_ composite system shows significantly improved kinetics in comparison to the pure Li-RHC system. In contrary to the dehydrogenation process of the pure Li-RHC, the plateau region is not observed in this sample. For both composite systems, the first reaction step's kinetics is very similar and is completed roughly after 0.25 h. However, the second reaction step of the doped system is improved by a factor of ∼20, relative to the pure Li-RHC system. To further investigate the influence of NbF_5_ on Li-RHC, coupled manometric- and DSC measurements were carried out on the pure and on the doped Li-RHC samples, and the results are shown in Fig. 2. The desorption curves were acquired using a manometric instrument in the coupled measurements confirm the results obtained by volumetric measurements. This is further confirmed by the analysis of the onset temperatures of the different peaks evolving during dehydrogenation. Apart from the first peak, due to the polymorphic transition of Li borohydride, that takes place at identical onset temperature in the two samples (113 °C), the onset temperatures of the other peaks are lower in the doped sample in comparison to the pure Li-RHC system (see Table 2). The temperature differences between the samples' peaks increase by moving from the second (Δ*T* = 21 °C) to the last (Δ*T* = 67 °C) one. Moreover, the dehydrogenation of the RHC is completed at 500 °C, with an induction time for the Li borohydride phase smaller than for the undoped sample. The peak with a small shoulder at lower *T* represents this step when the fluoride is present, instead of several less intense peaks (two visible before 500 °C in Fig. 2a). Concerning the second peak in the undoped sample, it is a sharp and single event. In the doped sample, it seems that a pre-melting takes place at lower *T* and an exothermic reaction occurs at the end of melting. This is coupled with a slow decrease of the sample mass, *i.e.* to a minor hydrogen release. The kinetics of this step is very slow. The reaction rate accelerates only when the Mg hydride dehydrogenation starts (under the 3 peak). By considering the dehydrogenation enthalpies, for MgH_2_ (3^rd^ peak), values of 61 ± 2 kJ mol^−1^ H_2_ and 58 ± 2 kJ mol^−1^ H_2_ are obtained for the undoped and doped sample, respectively, which means a slight destabilization due to the presence of NbF_5_. This value is in agreement with the values obtained in other studies under similar conditions.^47,48^ A more significant destabilization effect, however, is evident for the LiBH_4_ dehydrogenation enthalpy (4^th^ peak): at 4 bar the process is not completed for the undoped sample, while it is completed for the NbF_5_ containing system (characterized by an enthalpy of 13 ± 1.5 kJ mol^−1^ H_2_). This value is lower than the one reported for the dehydrogenation at 3 bar of the undoped system, *i.e.* 20 ± 3 kJ mol^−1^ H_2_.^47^

**Table 2:** Pure- and doped Li-RHC samples after first desorption with different isotopes

Sample/state	Pure Li-RHC	Description	Li-RHC + 0.1NbF_5_	Description
First-desorption	2LiH + MgB_2_	1D-L	2LiH + MgB_2_ + 0.1NbF_5_	1D-LN
	2LiH + Mg^11^B_2_	1D-11L	2LiH + Mg^11^B_2_ + 0.1NbF_5_	1D-11LN
	2^7^LiH + Mg^11^B_2_	1D-7L	2^7^LiH + Mg^11^B_2_ + 0.1NbF_5_	1D-7LN

**Fig. 3:**
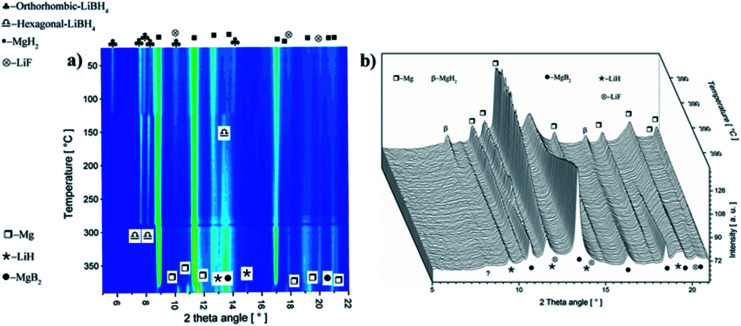
(a) *In situ* SR-XPD plot of the first desorption of the doped Li-RHC + 0.1NbF_5_ composite system collected at a temperature ramp of 3 °C min^−1^ up to a maximum temperature of 390 °C. (b) *In situ* SR-XPD plot at the isothermal condition (390°) and at 4 bar H_2_ backpressure.

To understand the effect of NbF_5_ on the reaction mechanism of Li-RHC, *in situ* SR-XPD was applied as a function of temperature. The result of these measurements is presented in Fig. 3. For the sake of a better overview, the *in situ* diffraction patterns are plotted in non-isothermal (Fig. 3a) and isothermal (Fig. 3b) conditions, respectively. Fig. 3a shows the *in situ* diffraction patterns of the doped sample from room temperature up to 390 °C (isothermal temperature). Intensities of the diffracted beam are plotted (in color code) over the temperature (in °C) and *2-theta* diffraction angle (in degrees ). As can be seen, the initial crystalline phases of the as-milled sample consist of MgH_2_, orthorhombic LiBH_4_ (o-LiBH_4_), and LiF. However, no diffraction peaks of any Nb-containing phase are detected. By raising the sample's temperature, simultaneous narrowing and shifting of the diffraction peaks are observed toward smaller angles. The narrowing of diffraction peaks can be inferred to a higher degree of recrystallization of the high-energy ball-milled material. The shift toward lower angles is due to the thermal expansion of the crystalline unit cells. The first change in the diffraction pattern occurs at about 120 °C. This is caused by the structural phase transformation of LiBH_4_ from orthorhombic o-LiBH_4_ (space group *Pnma*)^49^ to hexagonal h-LiBH_4_ (space group *P*6_3_*mc*),^50^ and matches with the first endothermal peak in the coupled volumetric-DSC measurement in Fig. 2b. At about 370 °C, the intensities of the Bragg-peaks of MgH_2_ start to diminish, and those of metallic Mg start to emerge. This corresponds to the second endothermic peak of the coupled volumetric-DSC curves, which is correlated to the dehydrogenation of MgH_2_ → Mg + H_2_. Shortly after that, diffraction peaks of MgB_2_ phase are detected. The immediate appearance of MgB_2_ after decomposition of MgH_2_ implies a reaction between metallic Mg and molten LiBH_4_, which corresponds to the third DSC signal in Fig. 2b. At isothermal conditions (Fig. 3b), the diffraction intensities of metallic Mg decreases rapidly, whereas the diffraction peaks of MgB_2_ and LiH are increasing correspondingly. This further confirms the assumption of a mutual reaction between metallic Mg and liquid LiBH_4_ phase. However, over the entire *in situ* SR-XPD measurement time, the state of niobium remains unknown. This could be due to the amorphous or/and nanoscale nature of Nb containing phase/s in the Li-RHC matrix.

**Fig. 4:**
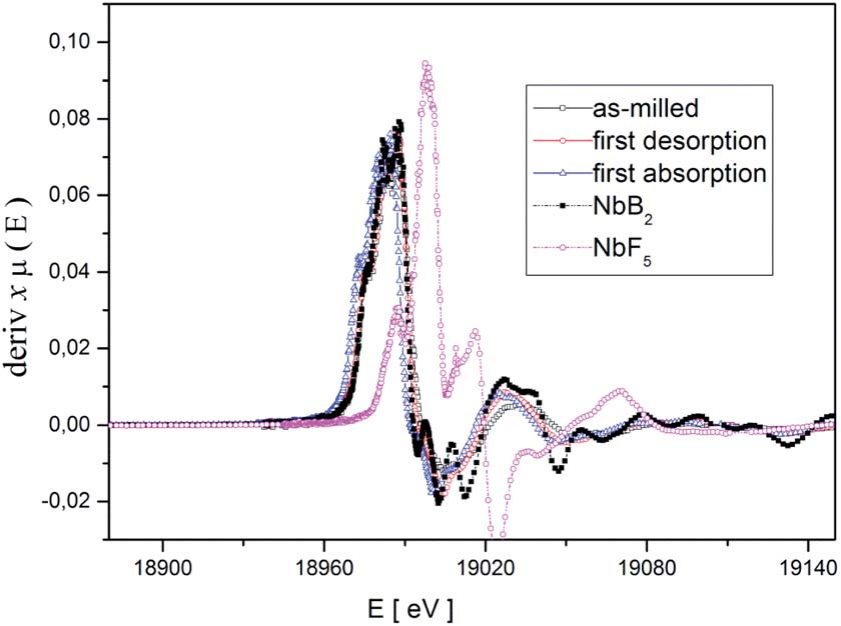
Derivatives of XANES spectra of the Li-RHC samples and NbF_5_ and NbB_2_ references.

**Fig. 5:**
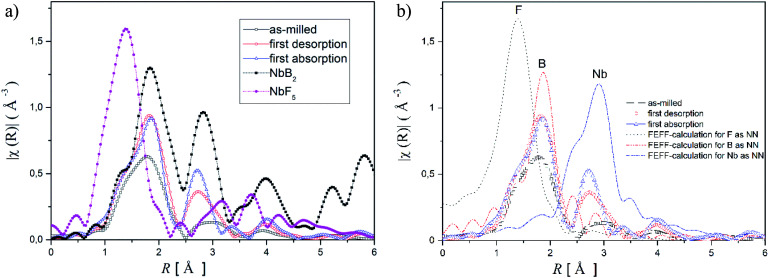
(a) Unweighted Radial Distribution Function (RDF) of Li-RHC samples and references in the *k*-range of 2–12 Å, FT from the EXAFS spectra, (b) RDFs of the samples and their corresponding calculated backscattering amplitude for the nearest neighbours for NbF_5_ and NbB_2_.

For obtaining detailed information about these phases, X-ray absorption spectroscopy (XAS) was applied. Because XAS method allows to detect elements in very small quantities in any environment, (in crystalline, or in an amorphous state). Hence, XAS measurements were carried out at the K-edge of niobium (18.98 keV) to determine the chemical state and the local environment of niobium in the hydride matrix at different hydrogenation cycle. Near edge structure of the XAS-spectra (XANES) was used to determine niobium's valance state in the Li-RHC system at different hydrogenation states. Additionally, pure NbF_5_ and NbB_2_ samples were measured as reference samples. In Fig. 4, the first derivatives of XANES of samples and references are presented. A comparison between the XANES spectra of the doped Li-RHC samples reveals that niobium has the same oxidation states at different hydrogenation states of the hydride matrix. On the other hand, a comparison between XANES spectra of samples and the NbB_2_ reference shows a great accordance in all details, which suggests an oxidation change in niobium valance from Nb^5+^ to Nb^2+^. To determine the local environment of Nb in the hydride matrix at different hydrogenation state, the EXAFS region of XAS spectra of the samples- and references (NbB_2_ and NbF_5_) were extracted and Fourier transformed (FT). The resulting radial distribution functions (RDF) are shown in Fig. 5a.

A comparison between the RDF patterns of the as-milled sample and the cycled samples shows that the local environment of Nb does not change upon dehydrogenation/hydrogenation procedure of the hydride matrix, after the milling process. Nevertheless, the width and size of the RDF amplitudes of the samples are differing at different cycling stages. However, their peak positions remain unchanged over the cycles. The RDF amplitudes of the samples increase with increasing hydrogenation cycles. This indicates that the degree of order around Nb (in the hydride matrix) is increased with increasing hydrogenation cycles of the sample. The comparison between the RDF patterns of the samples and NbF_5_ reference shows considerably changes in the local environment of Nb in the sample already after the ball milling process relative to NbF_5_ confirming the observation made by XANES.

**Fig. 6:**
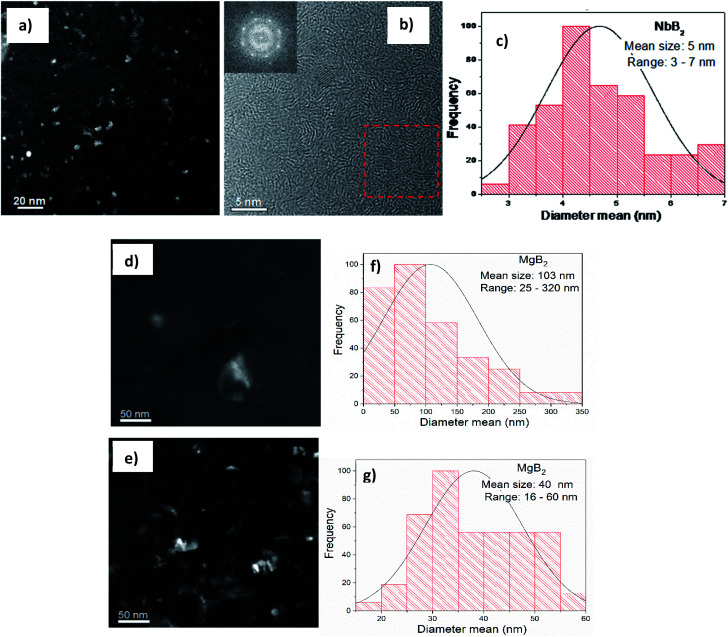
(a) TEM dark field image shows a homogeneous distribution of NbB_2_ particles. (b) Calculations based on inter-planar distances (*d*_*hkl*_ = 1/|*g*_*hkl*_|) of FFT images confirm the presence of NbB_2_ particles. (c) Calculated size distribution of NbB_2_ particles from TEM DF images. (d) DF image of MgB_2_ particles in the desorbed pure Li-RHC sample, (e) DF image of MgB_2_ particles in the desorbed doped Li-RHC sample. (f) Calculated size distribution of MgB_2_ particles from DF images of the desorbed pure Li-RHC sample. (g) Calculated size distribution of MgB_2_ particles of the desorbed doped Li-RHC sample.

**Fig. 7:**
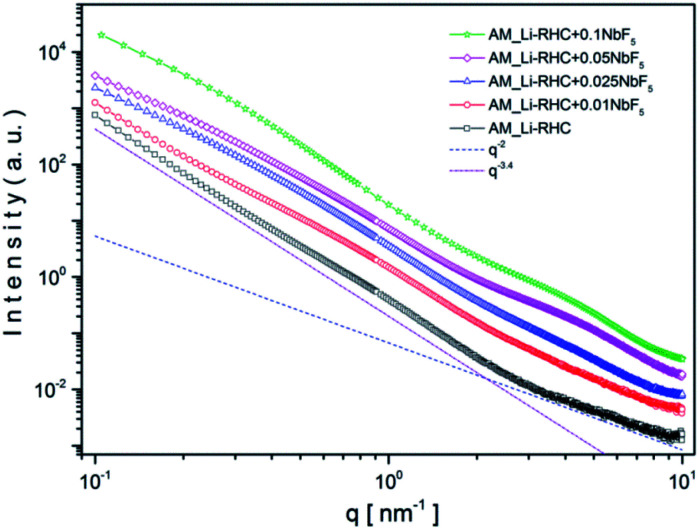
SAXS curves of as-milled Li-RHC sample with increasing NbF_5_ additive content (Li-RHC + *x*NbF_5_ with *x* being: 0, 0.1, 0.025, 0.05, 0.1 mol%) measured at 18.5 keV. Additional *q*^−*α*^ power-law lines are included to clarify the fractal character of the samples.

Comparing the RDF patterns of the samples and the RDF pattern of NbB_2_ reference shows an excellent agreement between them in overall details. This again strongly suggests the formation of NbB_2_ phase in the sample after the ball-milling process which remains stable upon cycling. To further confirm this observation, *ab initio* calculations were carried out using FEFF6 program.^38^ Results of these calculations and experimentally observed RFD patterns of the doped sample at different hydrogenation states are shown in Fig. 5b. In the calculation, the magnitudes of the scattering amplitudes were not included for electron relaxation processes since only the phase's existence was at the center of interest. FEFF-calculations were performed assuming NbF_5_ and NbB_2_ in crystalline state (space group *C*12/*m*1; Cryst. Sys. monoclinic (ICSD #26647)), (space group *P*6/*mmm*; Cryst. Sys. hexagonal (ICSD #30328)), respectively. As can be seen in Fig. 5b, the amplitude around 1 Å could be identified as the backscattering amplitude of fluorine. Moreover, the first- and second RDF amplitudes of the samples are well described by the backscattering amplitudes of boron and Nb, respectively. This confirms the formation of NbB_2_ phase in the as-milled and cycled Li-RHC + 0.1NbF_5_ samples. Therefore, Nb's local environment in the hydride matrix can be identified as NbB_2_. Further comparison between the RAD patterns of NbB_2_ reference and those of the samples shows that the RAD amplitudes of the samples drop significantly with increasing distance in comparison to those of the NbB_2_ reference. This indicates the presence of nanosized NbB_2_ crystalline particles in the samples, which explains the absence of any Nb containing diffraction signals in the *in situ* SR-XPD patterns above. To cross-check the chemical state of Nb containing phase(s) in the doped Li-RHC, and to investigate the microstructural effects of NbF_5_ on the hydride matrix, HR-TEM measurements were carried out. The results of these investigations are shown in Fig. 6. HR-TEM image of doped Li-RHC sample confirms the presence of nano-sized niobium-containing particles in the Li-RHC matrix. The length of each reflection (|*g*_*hkl*_|) could be measured using the FFT images (Fig. 6b) for these particles, and based on that, the inter-planar distances (*d*_*hkl*_ = 1/|*g*_*hkl*_|) could be calculated. The values for |*g*_*hkl*_| were in excellent agreement with those from the JEMS simulations for NbB_2_, thus confirming that for each case, indeed, NbB_2_ particles were formed. From dark field (DF) images, particle size measurements were carried out from which a local size distribution of NbB_2_ particles could be extracted (Fig. 6c). According to this distribution, the mean size of NbB_2_ particles is about 5 nm within the range between 3 and 7 nm. Additionally, HR-TEM micrographs (in DF mode) of MgB_2_ particles of pure- and doped Li-RHC samples in the desorbed state were taken (Fig. 6d and e). From the micrographs, size distributions of MgB_2_ particles in both samples were calculated, and the results are shown in Fig. 6f and g, respectively. As can be seen, the particle sizes of MgB_2_ in pure Li-RHC range from 25 up to 320 nm with an average particle size of 103 nm. In the doped Li-RHC sample, the corresponding particle sizes range from 16 nm up to 60 nm with a mean particle size of 40 nm. This clearly shows that the MgB_2_ particles in the doped Li-RHC sample (desorbed state) are much finer compared to the corresponding sizes in the Li-RHC sample. In order to characterize the size distribution of NbB_2_ particles in the hydride matrix in more detail, SAXS and ASAXS measurements were carried out. For SAXS measurements, samples with increasing NbF_5_ additive content (Li-RHC + *x*NbF_5_ with *x* being: 0, 0.01, 0.025, 0.05, 0.1) were prepared to investigate the effect of the additive on the hydride matrix. The results of SAXS measurements are shown in Fig. 7.

The *y*-axis represents the scattering intensities in arbitrary units, and the *x*-axis represents the scattering vector *q* in the inverse space (Å^−1^), where *q* = (4π/*λ*)sin *θ* and 2*θ* is the scattering angle. All SAXS measurements were carried out at the energy (18.54 keV) below the K-edge absorption energy of Nb (18.99 keV), to minimize the incoherent scattering. The SAXS curve of the sample without additive content (pure Li-RHC) exhibits a small and very broad shoulder around 1 nm^−1^ (which corresponds to structures of 4–7 nm in size), but otherwise, it is featureless over the entire observed *q*-range. This indicates a highly disordered material with a lack of morphological features over the measured size range. Nevertheless, the scattering curves can be divided into two main regions, which are well described by a simple *q*^−*α*^ power-law (dotted lines), respectively. This suggests a fractal morphology of the samples over the observed length scale.^51^ At the high *q*-region, between approximately 3 nm^−1^ and 10 nm^−1^, the power-law exponent is roughly *α* = 2, which corresponds to the scattering from a mass fractal structure. The length scale of these structures is between 1–3 nm, which is typical for distances in the grain boundaries. In the *q*-region between 0.1 nm^−1^ and 1 nm^−1^, the scattering curve follows a power-law behavior with an exponent of *α* = 3.4, which can be associated with scattering from particles with rough surfaces. The size scale of these particles lies in between 5 and 60 nm and larger. The fractal regions of the scattering curve gain morphological features with increasing NbF_5_ content in the Li-RHC sample. The very broad structure at the lower *q*-region around 0.3 nm^−1^ is observed first with an increment of 1 mol% NbF_5_ content. It becomes more prominent with increasing NbF_5_ content, while its peak center shifts toward lower angles and the width of its amplitude become extremely broad while its magnitude becomes larger. The second structure at higher *q*-region is centered around 3 nm^−1^ and becomes noticeable only after a NbF_5_ content of 2.5 mol%, but it becomes rapidly very pronounced with further addition of NbF_5_. In contrast to the first structure at the lower *q* region, the second structure becomes clearly visible only after adding of 5 mol% of NbF_5_.

This is due to the length scale of the structures in this region (1–3 nm) in comparison to structures found at the low *q* region (5–60 nm). Since the intensity of SAXS is ∼*V*^2^ ∼ *R*^6^ (where *V* is the volume, and *R* is the sphere's radius). Therefore, structures with relative small sizes are well discriminated by their SAXS intensities. It should also be noted that the overall fractal character of the scattering curves in both regions remain approximately valid. Interestingly, in the lower *q* region, the power-law decreases from 3.4 to roughly 2.9 with increasing NbF_5_ content. This indicates an increase in surface roughness (∼increase in surface area) of particles in this region with increasing NbF_5_ content. In contrast, the power-law exponent rises in the higher *q* region from 2.0 to approximately 2.5 with increasing NbF_5_ content, which implies a higher degree of compactness or increasing order of structures in this region with increasing NbF_5_ content. Although a systematic relation between NbF_5_ content and structures formed in the SAXS curves can be recognized, no specific statement about the size distribution of Nb-containing structures can be made due to the overlapping signal between the matrix and Nb containing structures. In order to extract the scattering intensities of Nb-containing structures, ASAXS measurements were carried out. Details about the ASAXS method can be found in the referred literature.^52–55^ One of the obtained results of an ASAXS measurement (Li-RHC + 0.1NbF_5_ after the milling procedure) is exemplary shown in Fig. S2a (ESI).† The peak scattering, located at around 4–5 nm^−1^, belongs to the scattering of the Kapton foil, which was used to seal the samples. In the inset figure, the sample's resonant behavior is emphasized with respect to variation in energy of the incident beam (near the K absorption edge of Nb). As the energy of the incident photons approaches the K absorption edge of the resonant atoms, the scattering intensities increase at the very high *q* region (Porod region) considerably. This effect is caused by fluorescence and inelastic Raman scattering of the resonant atoms, which leads to an additional (energy-dependent) constant scattering. However, the contribution of this constant scattering (relative to total structural scattering) becomes significant only in the Porod-region and at energies close to the absorption edge of a scattering element.^56^ Fig. S2b (ESI)† shows the background-corrected ASAXS scattering curves of Fig. S2a.† The shape of the scattering curves gives evidence of two structures in the sample (see inset figures). At the large *q* values of around 4 nm^−1^ the structures are rather resonant, and their estimated average size is approximately 2 nm (using the rule of thumb: *d* ≈ 2π/*q*). The structures located at about 0.3 nm^−1^ are less resonant in comparison to structures located at smaller *q* values, and their average size can be estimated to be around 10 nm. In order to isolate the pure resonant scattering of NbB_2_ structures from the total scattering, the Stuhrmann method (dispersion analysis) was applied (eqn (6)).^57^

6 *I*(*q*,*E*) = *I*_0_(*q*) + 2*f*′(*E*)*I*_0R_(*q*) + [*f*′^2^(*E*) + *f*″^2^(*E*)]*I*_R_(*q*)

**Fig. 8:**
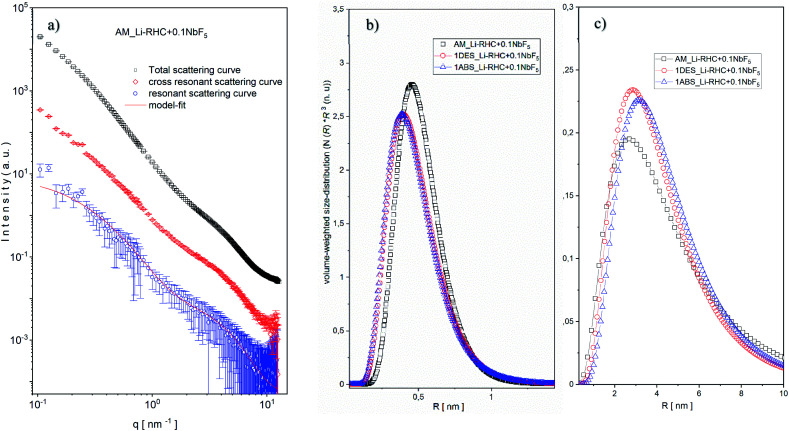
(a) Pure resonant scattering curve (blue circles) of as-milled sample and its model fit (red line), mixed resonant scattering curve (red diamonds), and the total scattering curve (black squares). Evolution of isolated bimodal size distributions of (b) small and (c) large NbB_2_ particles over a hydrogenation cycle of the doped Li-RHC system.

Here, *I*_0_(*q*) is the non-resonant scattering contribution to the overall scattering intensity, which equals the SAXS curve observed far from any resonant absorption edges. *I*_0R_(*q*) is the of-resonant (or mixed resonant) scattering contribution of superposed intensities of, both, resonant and non-resonant scattering. Using the dispersion analysis, the pure resonant scattering contribution of NbB_2_ structures could be isolated. In Fig. 8a, as an example, a result of this procedure is shown for the as-milled sample. As can be seen, the two structures discussed above are maintained in the pure resonant scattering curve, as well. Based on this fact, a bimodal spherical size distribution (eqn (1) and (2) in ESI†) was fitted to the pure resonant scattering curves (red line in Fig. 8a), assuming a lognormal distribution. The calculated bimodal size distributions for all samples are shown in Fig. 8b and c, respectively. As shown in Fig. 8b, the size of small NbB_2_ particles in the as-milled sample has an average size of ∼1 nm, which decreases slightly in number and in size (0.8 nm) after the first desorption and remains stable. In contrast to smaller particles, the bigger particles (Fig. 8c) grow after the first desorption in number and in size from 5 nm to ∼6 nm and remain roughly stable over the hydrogenation cycle. The bimodal size distribution and the evolution of small and larger NbB_2_ particles during cycling can be understood if one assumes that the NbB_2_ particles formed during the milling procedure are located in the grain boundaries of the hydride matrix as loosely linked mass fractals, as was suggested by SAXS results. During the 5 hours of high-energy ball milling, some clusters are merged to larger NbB_2_ nanoparticles (coarsening effect). That would explain the significantly lower number of larger NbB_2_ nanoparticles in the size range of 5 nm. Over the cycling procedure, some of the smaller NbB_2_ nanoparticles could migrate and agglomerate to/with larger particles; this can explain the decreasing amount of smaller NbB_2_ nanoparticles and the aggregate of larger NbB_2_ nanoparticles over the cycling procedure.

To study the structural effects of NbB_2_ nanoparticles on the hydride matrix (ultra) small-angle neutron scattering (SANS/USANS), measurements were carried out. For this reason, samples with and without additive after the first dehydrogenation process were measured. To determine the composition differences between particles of pure Li-RHC and doped Li-RHC + 0.1NbF_5_ systems, samples with different contrast were prepared by using lithium (^7^Li)- and boron (^11^B) isotopes, respectively. A list of samples for these measurements is given in Table 2.

**Fig. 9:**
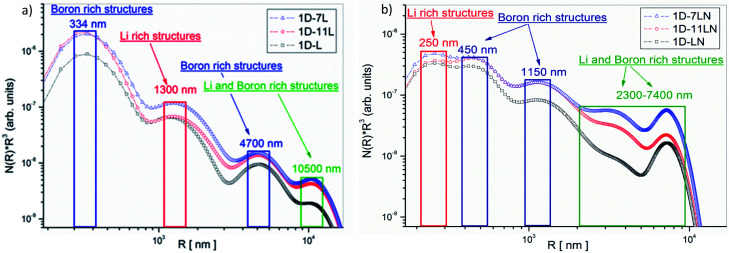
Size distribution of (a) pure Li-RHC and (b) doped Li-RHC + 0.1NbF_5_ with different isotopes after the first dehydrogenation reaction.

All samples were measured with SANS and USANS, respectively, in order to cover all matrix structures from the nm- to the µm-range in all samples. After calibration to absolute units, SANS and USANS curves were merged. An exemplary combined SANS/USANS result is shown in Fig. S3 (ESI).† To uncover the structural features depending only on the isotope effect, all SANS/USANS scattering curves of each sample with different isotopes were fitted simultaneously. Since contrast and number density of particles are directly correlated, only the number density of particles was fitted to pronounce the effect of isotope-containing structures. By applying this procedure and assuming spherical particles, size distributions of pure and doped Li-RHC systems were calculated. The calculated size distributions of pure and doped hydride matrices are presented in Fig. 9a and b. Both figures are plotted in a double logarithmic scale, where the *x*-axis represents the radii of spheres and the *y*-axis the volume-weighted number density of particles. The first observation of both size distributions shows that much smaller structures are present in the doped sample compared to the pure sample. Moreover, the size distributions of pure and doped Li-RHC sample, respectively, with different isotopes vary in their number densities. This can be attributed to different scattering length densities of the respective isotopes in each sample, which allow a qualitative interpretation of specific structures present in both isotope-containing samples. Due to higher coherent scattering and lower absorption cross-section of ^7^Li and ^11^B, the scattering intensities, hence the number density, of scattering structures in ^7^LiH–Mg^11^B_2_ and LiH–Mg^11^B_2_ should be comparable, in contrast to LiH–MgB_2_. Therefore, the size region around 334 nm (Fig. 9a) can be assigned to predominantly boron-containing structures. In the region at about 1300 nm the number densities of LiH–Mg^11^B_2_ and LiH–MgB_2_ samples are similar, however, smaller than the one of ^7^LiH–Mg^11^B_2_. This indicates that structures in this size range contain significantly less boron since the LiH–Mg^11^B_2_ and ^7^LiH–Mg^11^B_2_ samples have the same boron isotope. Thus, it can be deduced that these structures are predominantly lithium-containing structures. Likewise, it can be concluded that structures in the region at about 4700 nm are boron rich, whereas in the region at around 10 500 nm none of the size distribution curves cover each other entirely. Hence, it can be inferred that structures within this range are both lithium- and boron-containing structures. Size distributions of doped Li-RHC systems with different isotopes show a different behavior (Fig. 9b). The largest structures are much smaller (∼30%) in comparison to those found in the pure Li-RHC system. Moreover, for the above-mentioned reason, structures in the size range of 250 nm are predominantly lithium-containing, whereas structures at about 450 nm and 1150 nm are mainly boron rich. Particles in the range between 2300 nm and 7400 nm are lithium- and boron-containing, indicating a significantly better phase mixture in the Li-RHC + NbF_5_ sample in comparison to the pure Li-RHC sample.

## Discussion

**Fig. 10:**
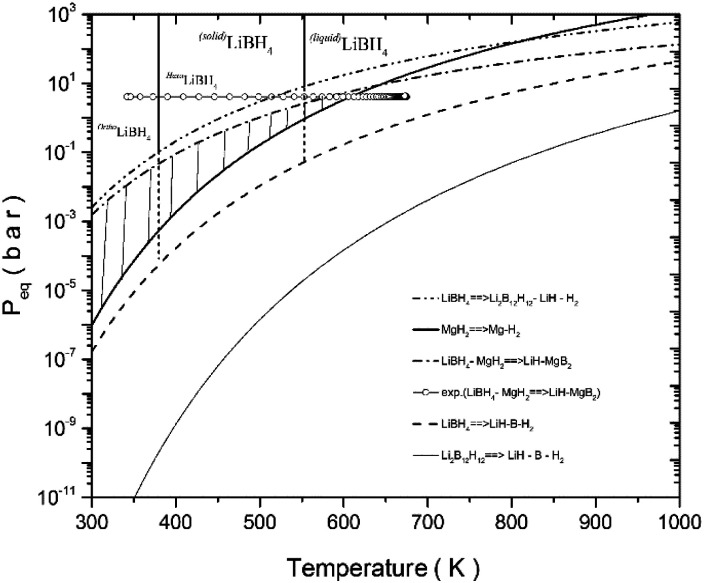
Phase diagram of orthogonal LiBH_4_, hexagonal LiBH_4_, liquefied LiBH_4_, Li_2_B_12_H_12_, MgH_2_, and LiBH_4_–MgH_2_ and LiBH_4_ with its different reaction paths. As an illustration, a volumetric desorption measurement of as-milled LiBH_4_–MgH_2_ sample. The hatched area displays the RHC phase. The values for enthalpy and entropy for the compounds and for the hydride composites were taken from ref. 30, ^31^ and ^58–62^.

Volumetric- and calorimetric measurements have shown that the addition of NbF_5_ to the LiBH_4_–MgH_2_ reactive hydride composite system enhances its dehydrogenation/rehydrogenation reaction kinetics by one order of magnitude, in comparison to its pristine state (see Fig. 1). Prior to its second dehydrogenation reaction step, the pure LiBH_4_–MgH_2_ composite system undergoes an extended plateau region where no reaction occurs. This plateau period, however, is not observed in the NbF_5_ doped system. Nonetheless, the dehydrogenation processes of both systems proceed in two steps. This can be explained by the phase diagram of LiBH_4_–MgH_2_, MgH_2_, and LiBH_4_, as is shown in Fig. 10. The experimental dehydrogenation reaction of Li-RHC is also plotted as supportive information, using volumetric measurements. The phase region, in which the composite system could react in one step to form LiH–MgB_2_, is restricted by the hatched area. In this region, the equilibrium pressure of the Li-RHC system is higher than that of both individual hydrides (LiBH_4_ and MgH_2_); hence, a one-step reaction is preferred. However, most of this dashed area lies in the solid phase region of LiBH_4_, where the reaction kinetic is highly constrained by mass diffusion and low surface contact area of both single hydrides. These kinetic limitations are minimized by applying experimental conditions where LiBH_4_ is in its liquid phase. The experimental conditions applied here lie outside the hatched area (open circles, see Fig. 10). Hence, the equilibrium pressure is lower than MgH_2_ but higher than LiBH_4_, which leads to a two-step reaction. Although the pure- and doped Li-RHC system follow a two-step dehydrogenation reaction, their reaction paths are very different, as shown by *in situ* SR-XPD measurements (see Fig. 3).

**Fig. 11:**
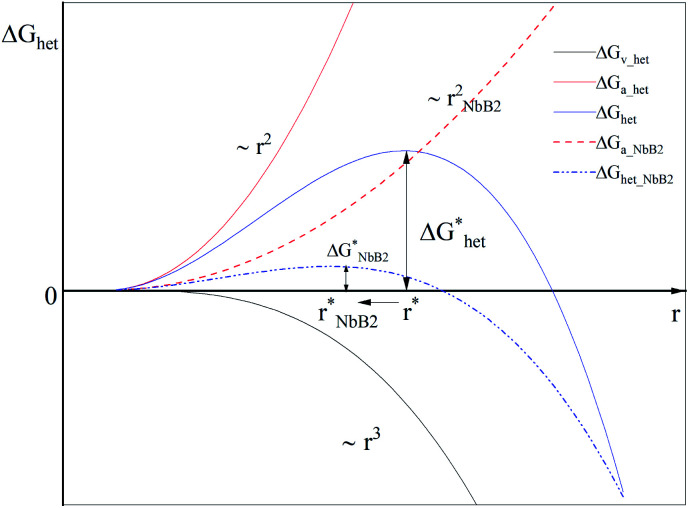
The change in Gibbs free energy required for heterogeneous nucleation for a spherical nucleus without- (blue solid line) and with NbB_2_ seeds (blue dashed-dotted blue line).

The doped Li-RHC follows a more complex reaction mechanism than the pure Li-RHC (reaction (4)). One difference is the formation of LiF in the as-milled state, which remains stable over the entire reaction path. The presence of LiF in the as-milled material also implies a reaction between NbF_5_ and the hydride matrix. However, no trace of any Nb containing phases could be detected by *in situ* XPD. Using the XAS, TEM, and ASAXS method, the chemical state and the local environment were determined, which indicated the presence of nano sized NbB_2_ particles, that remains stable over the dehydrogenation and rehydrogenation cycling processes. To understand the impact of NbB_2_ on the dehydrogenation/hydrogenation reaction mechanisms on the LiBH_4_–MgH_2_ composite system, kinetic models on volumetric data of the pure and doped systems are applied in order to find the rate-limiting steps for the desorption reaction of each system.^63–67^ Since the first desorption reaction step is the same for both systems, the second dehydrogenation reaction for both systems is examined using kinetic models listed in Table S1 (ESI).† For the fitting procedure, a transformed fraction of a maximum of 0.6 was chosen to describe the growth mechanism of MgB_2_ phase in both systems. The results of the transformed fraction *α*(*t*) of the pure Li-RHC and the doped Li-RHC + 0.1NbF_5_ systems are shown in Fig. S4 (see ESI†). Kinetics of both systems are well described by using a common model. The best fit suggests a two-dimensional growth of MgB_2_ phase with constant interface velocity of existing nuclei. The second best fit, which converges the curve with delay and diverges roughly two hours before the final state of the system, is obtained by a three-dimensional growth of MgB_2_ phase with constant interface velocity. Therefore, it can be concluded that the growth mechanisms of MgB_2_ in both systems are very similar. Based on these results, it can be assumed that the growth process of the MgB_2_ phase begins in a plate-like manner and at later stages the three-dimensional growth process becomes more dominant. Although the growth mechanisms of MgB_2_ in the pure and in the doped system are very similar, their respective rate conversion differs considerably. Therefore, it is worth focusing on the details of the second reaction step. After the decomposition of MgH_2_, metallic magnesium (Mg) remains in the solid phase, which is surrounded by the liquid phase of LiBH_4_. Thus, a reaction between these two phases can only occur at the solid–liquid interface by the formation of small nuclei of MgB_2_. However, the formation of these nuclei requires certain amount of energy, which has to be delivered by the system in order to initiate a heterogeneous nucleation and growth process of MgB_2_ phase. Here, the classical nucleation theory (CNT) can help to understand the underlying principles of nucleation and growth of MgB_2_ in this complex system as a very rough approximation. A very simple model for a heterogeneous nucleation process in CNT is given by a spherically shaped nuclei and its Gibbs free energy is:

7 Δ*G*_het_ = [−(4/3)πΔ*G*_v_*r*^3^ + 4π*γ*_a_*r*^2^]*S*(*θ*)

where, *r* represents the radius of the nucleus. Δ*G*_*v*_ stands for the energy decrease per converted unit volume, *γ*_a_ represents the increased interfacial energy per converted unit area between the nucleus and the matrix, and *S*(*θ*) is the shape factor, which describes the dependency of the “wetting” angle *θ* over the nucleus. For the sake of simplicity, it can be assumed that *S*(*θ*) ≈ 1. Then, eqn (7) (with its two contributors (solid lines)) can be plotted over the nucleus's radius, as shown in Fig. 11.

In the beginning, the nucleus is formed by a few atoms. Consequently, there are more atoms on its surface relative to its volume. Therefore, the nucleus bulk properties are dominated by surface characteristics (∼*r*^*2*^) of the nucleus. At a specific nucleus size, the volume properties (∼*r*^3^) of the nucleus start to determine its bulk properties. Therefore, the Gibbs free energy undergoes a maximum point 
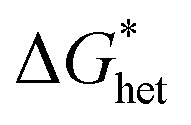
, where both effects are mutually balanced, and the corresponding *r*-value is denoted as “critical nucleus size”: r^*^. Therefore, nuclei formed with radii less than r^*^ are likely to decay, whereas nuclei with radii greater than r^*^ are more likely to overcome this energy barrier and remain stable with significantly higher probabilities. Consequently, the plateau phase in the pure Li-RHC system can be assigned to the nucleation period of MgB_2_ phase. NbB_2_ and MgB_2_ have the same hexagonal crystal structure. Furthermore, NbB_2_ provides two possible matching planes for the MgB_2_ phase to grow on, namely: MgB_2_ {1011} ‖ NbB_2_ {1011} and MgB_2_ {1010} ‖ NbB_2_ {1010} with *d*-mismatch values of 2.3% and 0.7%, respectively, which are well below the critical value of 6%.^68,69^ As was deduced from the SAXS/ASAXS results, NbB_2_ nanoparticles are distributed in the grain boundaries of the matrix and, therefore, NbB_2_ nanoparticles provide pre-existing stable nucleation centres for the formation of the MgB_2_ phase. This can lead to a significant reduction in the interfacial energy *γ*_a_ (see Fig. 11, dashed red line) and reduction in lattice strains between NbB_2_ and MgB_2_, promoting the nucleation and growth of MgB_2_ phase in the doped Li-RHC system. As a consequence, not only the energy barrier 
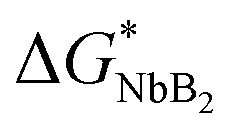
 for building stable NbB_2_ nuclei can be considerably reduced but also its corresponding critical nucleus size: 
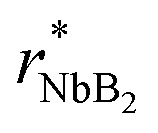
 (dashed-dotted blue line in Fig. 11), in comparison to pure Li-RHC. Since the thermodynamic driving forces are approximately the same in both systems, the volume contribution can be assumed to be comparable. Also, Mg provides a possible nucleation plane for MgB_2_ (MgB_2_ {0001} ‖ Mg {0001}); however, its directional misfit in the altitude 〈0001〉 of the hexagonal unit cell amounts up to about 48%. Taking into account that nucleation favorably does not occur on flat surfaces but along the ledges, this huge misfit (48%) would considerably increase the interfacial energy *γ*_a_, which in turn increases 
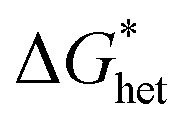
, and r^*^ hence hindering the nucleation and growth of MgB_2_ phase significantly. Moreover, since the formation of MgB_2_ nuclei is energy-consuming, the nucleation centers built in the pure Li-RHC system are scarcely and inhomogeneously distributed in the matrix compared to the doped system. This leads to a much slower reaction kinetics and larger MgB_2_ structures, which is observed in the pure Li-RHC system. In contrast, in the doped system abundant stable nuclei are provided (by the NbB_2_ nanoparticles) for nucleation and growth of MgB_2_. In addition, Zener pinning may also limit the growth of MgB_2_ structures in the doped system.^70^ Therefore, in the doped system much smaller MgB_2_ structures is expected, which is indeed observed by HR-TEM measurements. This is further supported by SANS/USANS results where the overall structures are observed to be smaller in the doped system in comparison to the pure system. This in turn creates significantly larger reaction surface areas in the doped Li-RHC and noticeably shorter diffusion paths relative to the pure Li-RHC system. The following schematic patch shows the key steps of the transformation of the additive, NbF_5_, and its role in improving the kinetic behavior of the Li-RHC:
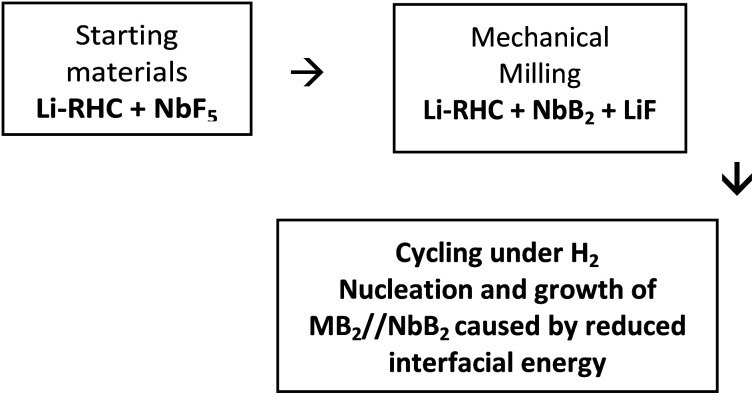


## Conclusions

In this study, LiBH_4_–MgH_2_ (Li-RHC) composite system was investigated. The dehydrogenation process of the pure LiBH_4_–MgH_2_ system proceeded in two steps which were separated by an incubation period and the overall reaction was completed after roughly 45 hours. The dehydrogenation/hydrogenation kinetics of this system could significantly be enhanced by an addition of small amount of NbF_5_.Here, the dehydrogenation process took place in reaction steps as well, however, the overall desorption reaction was completed after only 1.5 hours. Various experimental methods were applied to shed some light onto the catalytic function of NbF_5_ in dehydrogenation/hydrogenation reaction of Li-RHC system. X-ray absorption spectroscopy (XAS) investigation revealed that the local environment of NbF_5_ is changed during the milling process to NbB_2_, which remained stable upon further hydrogen desorption/absorption cycles. The results of SAXS/ASAXS investigation showed a nano-size distribution of NbB_2_ particles in the grain boundaries of the hydride matrix which are organized as loosely bounded clusters. The model also allowed concluding that NbB_2_ act as nucleation seeds for nucleation and growth of MgB_2_ phase, as no incubation period was observed in the doped system. Therefore, it was concluded that the presence of NbB_2_ nanoparticles considerably lowers the interfacial tension between the matrix and MgB_2_ nuclei, which can lead to a smaller energy barrier for the nucleation and growth process for MgB_2_ phase in the doped Li-RHC system. Indeed, noticeably smaller matrix structures were observed by SANS/USANS and high-resolution transmission electron microscopy (HR-TEM) measurements in the doped system in comparison to those in the pure Li-RHC system.

## Conflicts of interest

There are no conflicts to declare.

## Supplementary Material

RA-011-D1RA03246A-s001
