# Supplementary material for: A comprehensive study on lithium-based reactive hydride composite (Li-RHC) as a reversible solid-state hydrogen storage system toward potential mobile applications
Source: RSC Adv. 2021 Jun 30;11(37):23122–35. doi: 10.1039/d1ra03246a (PMC9034372; doi:10.1039/d1ra03246a)
Supplement: RA-011-D1RA03246A-s001 [file RA-011-D1RA03246A-s001.pdf]

Electronic Supplementary Material (ESI) for RSC Advances.
This journal is © The Royal Society of Chemistry 2021

Electronic Supplementary Information

A comprehensive study on Lithium-based reactive hydride composite (Li-RHC) as a reversible solid-state hydrogen storage system toward potential mobile applications

Fahim Karimi,*[a] Philipp Klaus Pranzas,[a] Julián Atilio Puszkiel,[c,a] María Victoria Castro Riglos,[b] Chiara Milanese,[d] Ulla Vainio,[e] Claudio Pistidda,[a] Goekhan Gizier,[a] Thomas Klassen,[a] Andreas Schreyer,[f] and Martin Dornheim[a]

[a]Department of Nanotechnology, Institute of Materials Research, Helmholtz–Zentrum Geesthacht, Max-Planck-Straße 1, 21502 Geesthacht, Germany.Address here.

[b]Department of Metalphysics, Consejo Nacional de Investigaciones Científicas y Técnicas (CONICET) y Centro Atómico Barilo-che, Av. Bustillo km 9500 S.C. de Bariloche, Argentina.

[c]Department of Physicochemistry of Materials, Consejo Nacional de Investigaciones Científicas y Técnicas (CONICET) y Centro Atómico Bariloche, Av. Bustillo km 9500 S.C. de Bariloche, Argentina.

[d]C.S.G.I. & Department of Chemistry, Physical Chemistry Section, University of Pavia, Viale Taramelli 16 - 27100 Pavia- ITALY.

[e]Hitachi High-Tech Analytical Science Finland, Finland

[f]European Spallation Source ERIC Box 176 S-22100 Lund, Sweden.

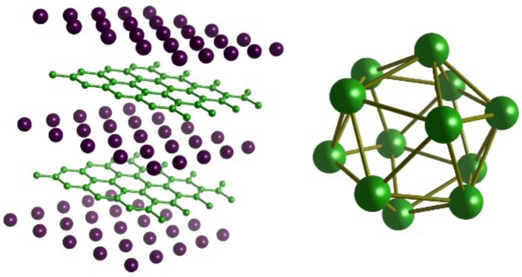

Figure S1. a) Layered structure of MgB_2 and b) Closo-structure of Boron Crystal structure.

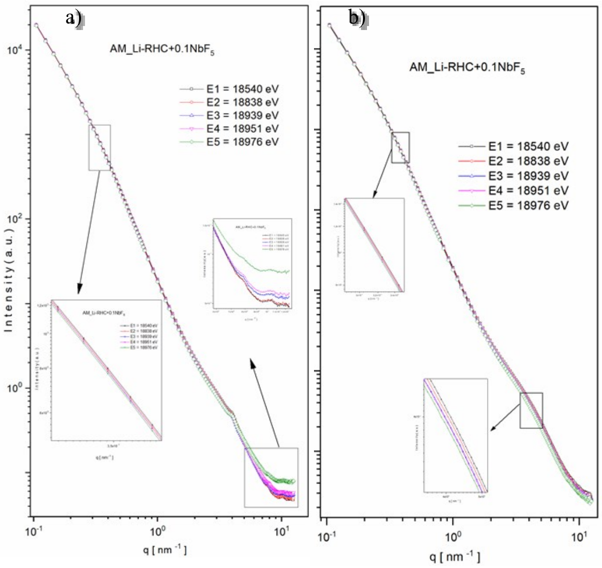

Figure S2. a) ASAXS curves collected at six energies close to the K-absorption edge of Nb (18.9 keV) b) ASAXS curves corrected for Kapton and incoherent scattering.

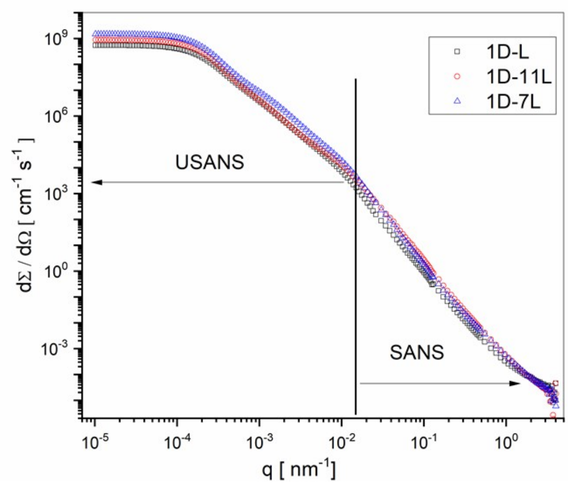

Figure S3. Merged SANS/USANS curves of pure $2LiBH_4+MgH_2$ with different isotopes after the first desorption.

Fitting model

For the SAXS/ASAXS and SANS/USANS scattering curves spherical particle distribution is assumed. The form factor of a sphere is given by:

$$F(q,r,\Delta\eta(E)) = 3\Delta\eta(E)\frac{\sin(qr) - qr\cos(qr)}{(qr)^3} \qquad (1)$$

and the size distribution of the spheres are assumed to be log-normal:

$$N(r) = \frac{1}{(2\pi)^2 r\sigma} exp\left(- \ln\left(\frac{r}{\mu}\right)^2 / 2\sigma^2 \right) \qquad (2)$$

Table S1. Kinetic models for fitting the volumetric measurements of pure- and doped Li-RHC.[1-3]

**Equation of the model**	**Model description**
$\alpha(t) = kt$	Chemisorption/recombination on the particle surface is the rate-limiting step.
$[-ln(1-\alpha(t)]^{1/2} = kt$	2-dimensional growth of existing nuclei with constant interface velocity (2D-KJMA).
$[-ln(1-\alpha(t)]^{1/3}) = kt$	3-dimensional growth of existing nuclei with constant interface velocity (3D-KJMA).
$[-ln(1-\alpha(t)]^{2/3}) = kt$	Diffusion controlled 3-dimensional growth of existing nuclei with decreasing interface velocity (DC: 3D-KJMA).
$1-(1-\alpha(t))^{1/2}) = kt$	2-dimensional growth of contracting volume with constant interface velocity (2D-CV).
$1-(1-\alpha(t))^{(1/3)} = kt$	3-dimensional growth of contracting volume with constant interface velocity (3D-CV).
$1-(2\alpha(t)/3)-(1-\alpha(t))^{2/3}) = kt$	Diffusion controlled 3-dimensional growth of contracting volume with constant interface velocity (DC: 3D-CV).

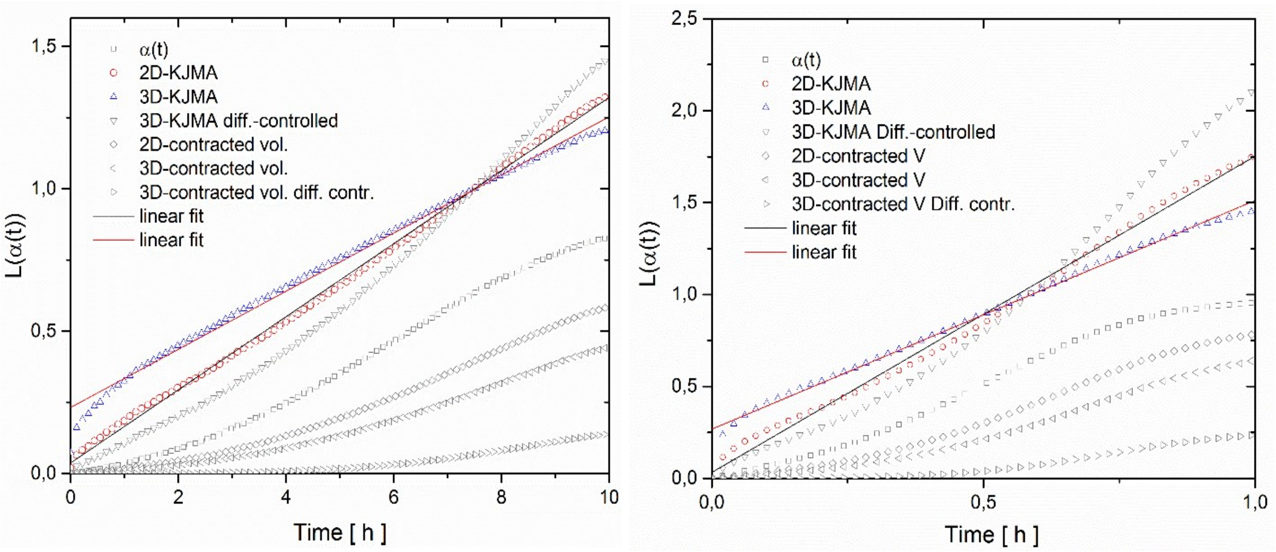

Figure S4. Kinetic models for the first desorption of a) pure Li-RHC and b) doped Li-RHC with the corresponding best fits.

References

1. M. Avrami, *The Journal of Chemical Physics*, 1939, **7**, 1103-1112.
2. M. Avrami, *The Journal of Chemical Physics*, 1940, **8**, 212-224.
3. M. Avrami, *The Journal of Chemical Physics*, 1941, **9**, 177-184.
